# Retraction statement: Down‐regulation of long noncoding RNA PVT1 inhibits esophageal carcinoma cell migration and invasion and promotes cell apoptosis via microRNA‐145‐mediated inhibition of FSCN1

**DOI:** 10.1002/1878-0261.13332

**Published:** 2022-12-02

The above article, published online on 01 August 2019 in Wiley Online Library (wileyonlinelibrary.com), has been retracted by agreement between the journal Editor‐in‐Chief, Kevin Ryan, FEBS Press, and John Wiley and Sons Ltd. The retraction has been agreed due to observed anomalies in the Western blots on figure 3B and D, the flow cytometry plots in figure 2C, and the wound healing assays in figure 7A and C. The authors were unable to provide compelling raw data underpinning figures 2, 3, 7, and 8.
